# Noninvasive Sensing of Foliar Moisture in Hydroponic Crops Using Leaf-Based Electric Field Energy Harvesters

**DOI:** 10.3390/bios16010013

**Published:** 2025-12-23

**Authors:** Oswaldo Menéndez-Granizo, Alexis Chugá-Portilla, Tito Arevalo-Ramirez, Juan Pablo Vásconez, Fernando Auat-Cheein, Álvaro Prado-Romo

**Affiliations:** 1 Departamento de Ingeniería de Sistemas y Computación, Universidad Católica del Norte, Antofagasta 1249004, Chile; hernan.chuga@alumnos.ucn.cl (A.C.-P.); alvaro.prado@ucn.cl (Á.P.-R.); 2 Department of Electrical Engineering, Pontificia Universidad Católica de Chile, Santiago 7820436, Chile; tito.arevalo@uc.cl; 3 Department of Mechanical Engineering and Metallurgy, Pontificia Universidad Católica de Chile, Santiago 7820436, Chile; 4 Energy Transformation Center, Faculty of Engineering, Universidad Andres Bello, Santiago 7500971, Chile; juan.vasconez@unab.cl; 5 Department of Engineering, Harper Adams University, Edgmond, Newport TF10 8NB, UK; fauat@harper-adams.ac.uk

**Keywords:** electric field energy harvesting, wearable devices, smart agriculture, sensor systems and applications, vegetation

## Abstract

Large-scale wireless sensor networks with electric field energy harvesters (EFEHs) offer self-powered, eco-friendly, and scalable crop monitoring in hydroponic greenhouses. However, their practical adoption is limited by the low power density of current EFEHs, which restricts the reliable operation of external sensors. To address this challenge, this work presents a noninvasive EFEH assembled with hydroponic leafy vegetables that harvests electric field energy and estimates plant functional traits directly from the electrical response. The device operates through electrostatic induction produced by an external alternating electric field, which induces surface charge redistribution on the leaf. These charges are conducted through an external load, generating an AC voltage whose amplitude depends on the dielectric properties of the leaf. A low-voltage prototype was designed, built, and evaluated under controlled electric field conditions. Two representative species, *Beta vulgaris* (chard) and *Lactuca sativa* (lettuce), were electrically characterized by measuring the open-circuit voltage (VOC) and short-circuit current (ISC) of EFEHs. Three regression models were developed to determine the relationship between foliar moisture content (FMC) and fresh mass with electrical parameters. Empirical results disclose that the plant functional traits are critical predictors of the electrical output of EFEHs, achieving coefficients of determination of $R^{2}=0.697$ and $R^{2}=0.794$ for each species, respectively. These findings demonstrate that EFEHs can serve as self-powered, noninvasive indicators of plant physiological state in living leafy vegetable crops.

## 1. Introduction

Crop security has become a fundamental pillar of sustainable agricultural production, requiring noninvasive and real-time monitoring of plant health to prevent stress-induced yield losses [1]. Conventional imaging-based techniques, such as multispectral and hyperspectral cameras, enable crop phenotyping and provide important information about plant physiology and health [2,3]. However, their high cost, limited spatial resolution, and strong dependence on ambient lighting conditions limit their effectiveness in controlled hydroponic environments [4,5]. To address these challenges, recent advances in the Internet of Things (IoT) have led to the development of wearable and distributed devices for continuous crop monitoring [6]. Despite their potential, large-scale deployments face several challenges related to data security, data management, and environmental sustainability [7,8,9]. Furthermore, the dependence on lithium-based batteries presents an important concern, highlighting the need for self-powered crop security systems [10].

To address these issues, displacement-current-based energy harvesters (DC-EHs) have emerged as promising, eco-friendly power sources for self-powered agricultural sensors [11]. In particular, DC-EHs have received increased attention for the development of self-powered active sensors because of their high power density and the abundance of exploitable ambient energy sources [12]. Recent research efforts have focused on the manufacturing of DC-EHs using recyclable and biodegradable natural materials [11,13]. Most of these studies have concentrated on replacing conventional dielectric and metal layers with different plant organs, such as leaves, stems, or fruits, demonstrating the capability of natural tissues as functional materials for green energy harvesters [14,15,16,17,18]. Preliminary works have demonstrated the harvesting capabilities of leaf-based electrodes, achieving power densities of up to 6 μW × cm^−2^, which are used to drive low-power IoT sensors [15,19,20]. However, the correlation between plant functional traits and the electrical performance of green DC-EHs is still poorly understood.

In 2018, Y. Jie et al. introduced a functional prototype that achieved 45 mW/m^2^ of power density, which energizes LEDs and portable electronics [19]. Liquid–solid contact electrification in natural leaves, specifically for Lotus leaves, was undertaken in [20]; the DC-EH achieved 6 μW/cm^2^ by exploiting the friction between the leaf surface and water drops. With the same aim, a fully biodegradable harvester was designed, assembled, and tested in [21]. Other outstanding works have disclosed the ability of DC-EHs to harvest energy from wind [22,23]. Fallen leaves have also been used as electrodes for TENGs [24], achieving power outputs of up to 8.02 μW/cm^2^. In addition, electrodes can be assembled using plant stems and soil [25]. Furthermore, in [14], the authors demonstrated that the high water content in vegetables enables the development of fully biodegradable TENGs. With the same aim, Zhang et al., in 2022, reported that other vegetable species, such as onion, leek, and scallion, exhibit high power density when used as electrode materials in TENGs. Recently, a self-powered humidity sensor was proposed in [16]; the proposed sensor monitors plant humidity and power with a TENG assembled with the same leaf. A large number of published studies describe the development of artificial trees based on Leaf-TENG, which can be used for IoT in agriculture [16,18,22,25,26]. Recent evidence suggests that leaf-EHs can also harvest environmental energy through electromagnetic radiation [17]. This view is supported by F. Merder et al., who determined that plant-hybrid EHs can harvest environmental kinetic energy using mechanical movements or even radiofrequency radiation [18].

In addition to TENGs, several DC-EHs have recently emerged for smart agriculture, including soil electrode-potential harvesters that exploit natural electrochemical gradients in plant substrates [27], microbial fuel cells that convert rhizosphere biochemical activity into stable DC output [28], and displacement-current harvesters that use hydrated plant tissues as bio-dielectric media for self-powered phenotyping [29]. Complementary self-powered sensing technologies demonstrate the increasing feasibility of energy-autonomous systems in precision agriculture. Among the technologies are graphene-based stretchable strain sensors for in situ plant monitoring [30], wearable multimodal platforms for continuous physiological detection [31], and hydrogel-based multimodal direct current sensors. Furthermore, emerging Internet of Drones (IoD) frameworks highlight the need for long-lasting, self-sustaining sensing nodes in large-scale agricultural monitoring [8].

Although extensive research has been conducted on eco-friendly DC-EHs, preliminary works have mostly focused on evaluating their electrical performance based on the technical characteristics of external energy sources—such as the frequency of mechanical oscillations for TENGs or the mains frequency and voltage for electric field energy harvesting (EFEH) [11]. In general, DC-EHs have been used as the energy source to energize low-power electronics and wearable devices. Only a few studies have presented a comprehensive analysis of green DC-EH performance according to the plant characteristics. More research is needed to understand the relationships between the performance of harvesters and the plant phenotyping [14,16,17,29]. Establishing such correlations is essential for developing self-powered sensors capable of determining physiological conditions through their electrical response.

This work presents the analysis of the energy harvesting and sensing capabilities of leaf-based EFEHs assembled with hydroponic leafy vegetables. Two varieties of hydroponic species, *Beta vulgaris* and *Lactuca sativa*, are experimentally characterized by measuring the open-circuit voltage (VOC) and short-circuit current (ISC) under controlled electric field conditions. The collected data is analyzed using three regression models to determine how the fresh mass and foliar moisture content impact EFEH electrical performance.

The rest of this work is organized as follows. Section 2 introduces the experimental methodology, including EFEH assembly, controlled dehydration, electrical measurements, and data processing. Section 3 presents the electrical characterization results and their correlations with plant functional traits. Section 4 analyzes the underlying mechanisms, species-dependent behavior, and implications for self-powered plant monitoring. Finally, Section 5 presents the conclusions drawn from this work.

## 2. Materials and Methods

**Figure 1:**
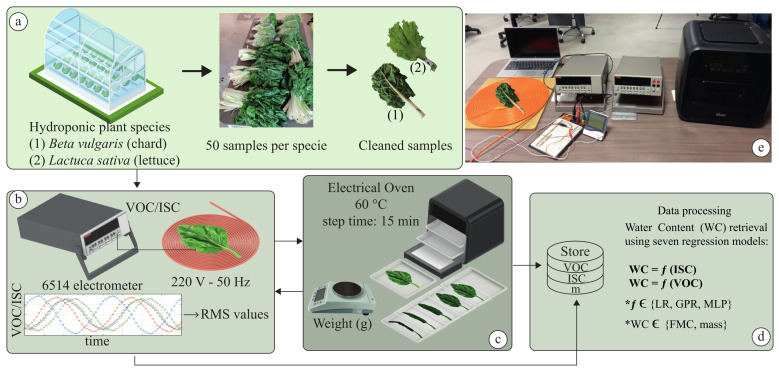
Representative diagram of EFEHs assembled with vegetables, as proposed in this work. The proposed methodology consists of four main stages: (**a**) harvesting and preparation of raw plant material; (**b**) EFEH assembly with freshly harvested hydroponic species, followed by sampling of electrical parameters (VOC and ISC) and fresh mass at several dehydration stages; (**c**) controlled dehydration of each sample under laboratory conditions to analyze the relationship between electrical parameters and plant moisture content; and (**d**) use of the collected dataset to construct different regression models. (**e**) Experimental setup. Note: The asterisks (*) indicate the machine learning model and the moisture parameter being evaluated. In this work, Linear Regression (LR), Gaussian Process Regression (GPR), and a Multilayer Perceptron (MLP) are used. In addition, two target parameters are considered: foliar moisture content (FMC) and mass.

This section introduces the experimental design, sample collection, and measurement processes used to analyze the electrical behavior of leaf-EFEHs. As shown in Figure 1, the proposed methodology comprises four main stages: (a) collection and preparation of samples, (b) assembling of EFEHs and electrical characterization, (c) dehydration and plant functional traits measurement, and (d) data-driven analysis using regression models. All experiments were conducted under controlled laboratory conditions to ensure reproducibility.

### 2.1. Operational Principle of Leaf-EFEH

Capacitive coupling refers to the transfer of energy in a time-varying electrical system through the displacement current between conductive elements separated by a dielectric medium. The displacement current is computed as

(1) $$\begin{array}{c} I_{d}=\int_{S}\frac{\partial \vec{D}}{\partial t}\cdot d\vec{S}, \end{array}$$

where $\vec{D}$ is the electric displacement field, $d\vec{S}$ is the differential surface element, and *t* denotes time.

The leaf-based EFEH requires a time-varying electric field to operate. An alternating voltage source produces a periodically varying electric field that induces charge redistribution on nearby conductive devices. A conventional EFEH consists of three elements: a floating electrode, a dielectric medium, and a grounded reference electrode [10].

In the proposed device, the leaf fulfills a dual role: (i) it acts as a bio-dielectric medium whose electrolyte-rich structure determines the effective coupling capacitance, and (ii) its ionic gradients and conductive pathways contribute to the internal impedance of the equivalent circuit. Variations in foliar moisture content modify both the permittivity and the resistive component of the leaf–electrode interface, producing quantifiable changes in VOC and ISC. The cable jacket of the measurement lead acts as the dielectric medium, while a grounded metal bar is used as the reference electrode.

### 2.2. Sample Collection and Preparation

This work analyzes how plant functional traits affect the electrical performance of EFEHs assembled with hydroponic vegetable species. Large, healthy, and mature leaves were collected from hydroponic species cultivated in Antofagasta, Chile, between 12 September 2024 and 30 September 2024. In this context, two representative leafy vegetables, *Beta vulgaris* and *Lactuca sativa*, were studied due to their high water content, broad availability, and consistent morphology. A set of 100 samples (50 samples per species) were harvested, ensuring biological variability by selecting one leaf per plant. The samples were maintained in an eco-friendly enclosure designed to safeguard them from physical damage and adverse environmental conditions [32]. Before assembly, all leaves were cleaned with deionized water to remove impurities and dried at 20 °C and 50% relative humidity for 1 h to stabilize surface moisture—conditions aligned with ISO/IEC calibration standards [33]. These parameters ensured repeatable electrical contact and avoided surface condensation effects.

### 2.3. EFEH Fabrication and Measurements

Each EFEH was fabricated by replacing one metal electrode with a natural leaf, forming a DC-EH configuration operating under the EFEH mode. As shown in Figure 1, a metallic plate was attached to the leaf surface to work as the signal–electrode interface between the plant tissue and grounded plate. A metallic bar was connected to the ground, completing the leaf-EFEH topology used in this work. The noninvasive configuration guaranteed charge transfer while avoiding mechanical damage to the leaf cuticle.

A stable alternating electric field (220 V, 50 Hz) was generated using a 24 m three-wire cable (hot, neutral, and ground), following the setup described in [34]. The electrical behavior of EFEHs was characterized by measuring VOC and ISC, as shown in [10]. Both parameters were recorded using a Keithley 6514 electrometer (Keithley Instruments, Cleveland, OH, USA) with high input impedance (≥200 TΩ) [29]. The recorded data were transmitted to a workstation using a Keithley KUSB-488B USB–GPIB interface (Keithley Instruments, Cleveland, OH, USA) at a sampling rate of 620 samples per second [35]. Data logging and analysis were performed in MATLAB 2023b on a high-performance workstation.

### 2.4. Controlled Dehydration Process

To analyze the correlation between the electrical outputs and plant functional traits, each EFEH was subjected to a controlled dehydration process [6,29]. In particular, leaf fresh mass and foliar moisture content (FMC) were studied. Leaves were dried using an Oster industrial dehydrator at constant temperature and airflow, following the procedures in [36,37]. For each species, optimal drying parameters were empirically determined, while ensuring continuous moisture loss and preserving structural integrity.

The mass of the EFEH was measured at each dehydration stage using a 0.01 g Biobase BP12002 precision balance (±0.01 g). The foliar moisture content (FMC) was computed as follows:

(2) $$FCM\left(\%\right)=\frac{W_{f,t}-W_{d}}{W_{f,t}}\times 100\%,$$

where $W_{f,t}$ and $W_{d}$ are fresh and dry mass, respectively. In addition, a 16-megapixel monocular camera was used to capture a photo of each sample. At each dehydration stage, electrical parameters were stored until the leaf achieved the moisture equilibrium.

### 2.5. Data Analysis and Regression Models

The dataset consisted of tuples of measurements (VOC, ISC, mass, and FCM) for both species. The first two variables correspond to six time-domain signals containing 620 samples each, from which the root mean square (RMS) value was calculated. The mean of the RMS values across repetitions was then used as the representative electrical feature for each sample. Three regression models were implemented to estimate plant traits from the electrical data: linear regression (LR), Gaussian process regression (GPR), and a multilayer perceptron (MLP). The three proposed models were used due to their outstanding performance in estimating plant functional traits [29,38,39]. Table 1 summarizes the main hyperparameters of the proposed regression models used in this work.

To ensure a proper and unbiased comparison between the electrical parameters of the EFEH and plant functional traits, we applied a k-fold cross-validation during the training process. All the samples were randomly ordered and divided into k groups. In our case, k was selected as 10. For each fold, one of the ten groups was used for testing, while the remaining data served as training data. The evaluation metrics were calculated based on the results obtained from the testing data. This cross-validation method increases the model’s reliability by assessing its performance on multiple subsets.

## 3. Experimental Results

This section analyzes the electrical performance of EFEHs assembled with hydroponic leaves, evaluates the three regression models for predicting FMC and fresh mass, and validates their potential as low-power energy harvesting devices.

### 3.1. Electrical Performance Evaluation Under Variable Resistors

**Figure 2:**
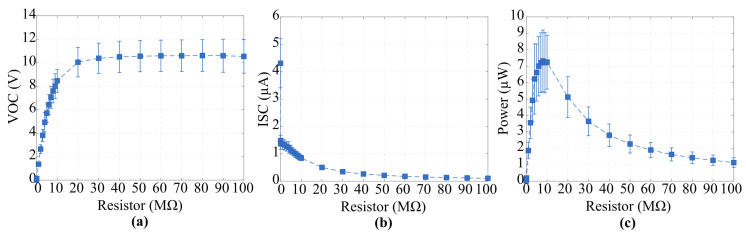
Electrical response of the leaf-based EFEH assembled with Beta vulgaris under different external load resistances: (**a**) open-circuit voltage, (**b**) short-circuit current, and (**c**) harvested power. A maximum power point is observed at approximately 10 MΩ, consistent with the Thevenin-equivalent behavior of the EFEH as a finite current source.

To quantify the effective power that can be harvested from the leaf-based EFEH, we measured its electrical output across external load resistances ranging from 1 kΩ to 100 MΩ, following the methodology described in [40]. Figure 2 shows the resulting VOC, ISC, and power curves obtained for *Beta vulgaris* under identical electric field excitation conditions. As expected for a finite-current source, the output voltage increases with load resistance while the current decreases, yielding a characteristic power–load curve with a single maximum [41]. The harvested power presents an MPP at approximately 10 MΩ, where the device achieves a peak output of 7.25 μW, with a corresponding open-circuit voltage of 8.45 V and a short-circuit current of 846 nA. Although the absolute power levels are in the same order of magnitude as those reported for state-of-the-art bio-based and moisture-driven energy harvesters, identifying this MPP is crucial for optimizing impedance matching in ultra-low-power circuits and confirms that the leaf-based EFEH can deliver practical and usable energy under realistic operating conditions.

### 3.2. Electrical Performance Evaluation

**Figure 3:**
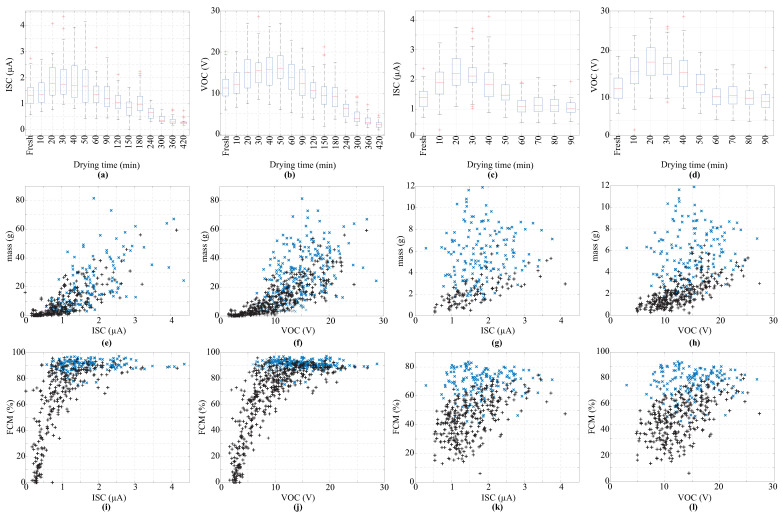
Electrical characterization of EFEHs assembled with two species of leafy vegetables: *Beta vulgaris* and *Lactuca sativa*. (**a**,**b**) The behavior of drying time versus ISC and VOC for *Beta vulgaris* species. (**c**,**d**) The behavior of drying time versus ISC and VOC for *Lactuca sativa* species. (**e**,**f**) Electrical parameters (ISC and VOC) versus mass for *Beta vulgaris* species. (**g**,**h**) Electrical parameters versus mass for *Lactuca sativa* species. (**i**,**j**) Electrical parameters (ISC and VOC) versus FMC for *Beta vulgaris* species. (**k**,**l**) Electrical parameters versus FMC for *Lactuca sativa* species. Note: Black plus markers indicate measurements taken before reaching the MPP, while blue “x” signs represent measurements taken after the MPP.

The electrical performance of EFEHs was characterized by analyzing the relationships between VOC and ISC as functions of drying time, fresh mass, and FMC. Figure 3 summarizes the key findings. On average, EFEHs assembled with *Lactuca sativa* leaves outperformed those assembled with *Beta vulgaris* by up to 10 % in VOC. Freshly harvested *Lactuca sativa* samples exhibited a median VOC of 12.3 V and an ISC of 1.38 μA, compared with 11.19 V and 1.33 μA for *Beta vulgaris* leaves. Although *Beta vulgaris* presented consistent behavior across different samples, a few samples exhibited the highest electrical performance; for instance, Sample 12 produced 20.06 V and 2.75 μA, exceeding maximum values observed for *Lactuca sativa* leaves (19.33 V and 2.37 μA).

Across the 100 samples, a common two-stage electrical pattern was observed: both VOC and ISC increased during the early dehydration stage, peaked at a maximum power point (MPP), and then decreased continuously. *Lactuca sativa* reached up to 18.22 V and 2.21 μA after 20 min of drying at 60 °C. On the other hand, *Beta vulgaris* leaves achieved 15.91 V and 1.74 μA after 50 min under the same conditions. After the MPP was achieved, both parameters decreased as dehydration advanced. *Lactuca sativa* exhibited a faster reduction in ISC and VOC due to the thinner and less moisture-retentive leaf structure. These findings suggest that EFEHs assembled with *Beta vulgaris* leaves could provide extended operational lifespans due to their high resistance to dehydration.

Leaf mass, directly associated with water content, was the first plant functional trait analyzed. Figure 3e,f present the electrical performance for *Beta vulgaris* samples, while Figure 3g,h show the corresponding results for *Lactuca sativa*. Before the MPP, VOC and ISC increased as leaf mass decreased (see blue × markers). By contrast, both electrical parameters exhibit an inverse trend after reaching the MPP (see black + markers). The reduction was more pronounced for ISC. Leaf mass, directly associated with water content, was the first plant functional trait analyzed. Figure 3e,f show the electrical behavior for Beta vulgaris and Figure 3g,h for Lactuca sativa. Before the MPP, both VOC and ISC increased as leaf mass decreased (blue × markers), while after the MPP they followed an inverse trend (black + markers). The reduction was more pronounced for ISC, whereas VOC maintained a steadier, approximately linear correlation with mass—indicating that VOC is a more reliable predictor. Moreover, *Beta vulgaris* data exhibited lower dispersion than *Lactuca sativa*, reinforcing its suitability for self-powered sensing. The higher variability observed before the MPP implies that regression-based ML models may perform poorly during early dehydration but improve under advanced drying stages. These results highlight that EFEH-based active sensors are most effective for monitoring plant functional traits under high-water-stress conditions, where strong correlations emerge for both species.

Finally, we analyzed potential correlations between electrical parameters and FMC, as shown in Figure 3i–l. As expected from previous results, a dual performance is shown before and after the MPP. Unlike the relationship with leaf mass, this plant functional trait presents an exponential trend in both VOC and ISC for samples under high water stress. In addition, FMC functionals are relatively constant at early stages of drying (before the MPP). Although the leaf has begun losing water, the electrolytes are still dissolved and evenly distributed, thereby preserving the electrical behavior. These results support the idea that active sensors are more suitable for use during advanced stages of dehydration or water stress, when clearer electrical correlations appear. Moreover, the observed increase in VOC and ISC during initial drying stages (refer to Figure 3a–d) is related to the water loss from the leaf surface, which enhances the connection between the alligator clips and the internal electrolytes through the stomata. It is worth noting that this plant functional trait presents greater variability in *Lactuca sativa* samples compared to *Beta vulgaris*. This finding suggests that while *Lactuca sativa* may be suitable for collecting electrostatic charge, it is less reliable for use in estimating plant functional traits through electrical outputs of the EFEH.

### 3.3. Performance Evaluation of Regression Models for *Beta vulgaris*

As shown in Table 2, the performance evaluation of the three proposed regression models (LR, GPR, and MLP) for predicting leaf mass and FMC in *Beta vulgaris* species reveals that clear differences exist before and after reaching the MPP. For leaf mass estimated from the VOC before the MPP, the GPR model had the best performance, with an RMSE of 15.39 and a R^2^ of 0.338. However, the three regression models presented poor performance in this process, verifying the high variability of the data during early leaf dehydration. By contrast, during the second stage (after the MPP), the three models showed improvements in their performance. The GPR model presented the best fit, achieving an RMSE of 6.37 and R^2^ of 0.698. The MLP models also achieved outstanding performance, with an RMSE of 6.41 and R^2^ of 0.694. Empirical findings disclosed that there is a strong linear correlation between foliar mass and VOC in high-water-stress conditions.

**Figure 4:**
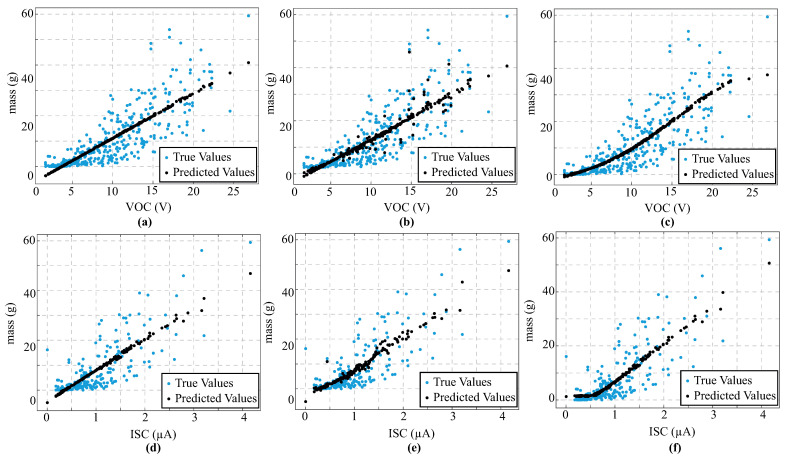
Performance evaluation of different regression models to predict leaf mass using electrical parameters—VOC and ISC. (**a**) Linear regression (LR) using VOC. (**b**) Gaussian process regression (GPR) using VOC. (**c**) Multilayer perceptron (MLP) using VOC. (**d**) Linear regression using ISC. (**e**) Gaussian process regression using ISC. (**f**) Multilayer perceptron using ISC. Note: Cyan point markers indicate the measured values, while black point markers represent the predicted values from each regression model.

**Figure 5:**
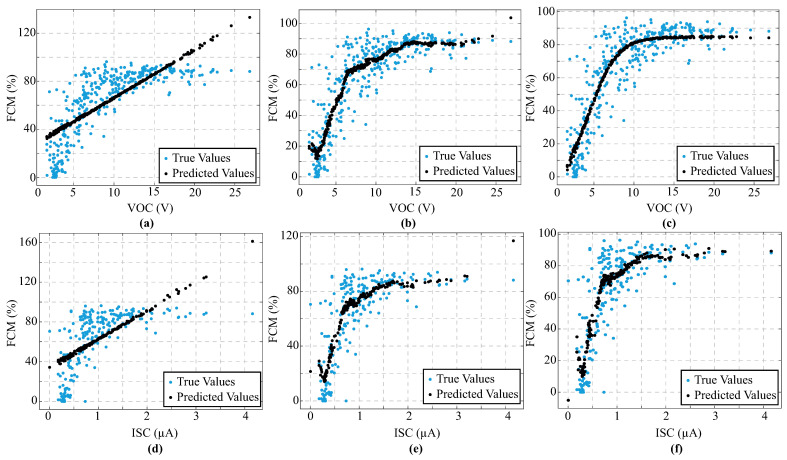
Performance evaluation of different regression models to predict FMC using electrical parameters—VOC and ISC. (**a**) Linear regression (LR) using VOC. (**b**) Gaussian process regression (GPR) using VOC. (**c**) Multilayer perceptron (MLP) using VOC. (**d**) Linear regression using ISC. (**e**) Gaussian process regression using ISC. (**f**) Multilayer perceptron using ISC. Note: Cyan point markers indicate the measured values, while black point markers represent the predicted values from each regression model.

On the other hand, GPR outperformed others regression model in terms of predicting leaf mass using ISC values acquired prior to the MPP, achieving an RMSE of 9.54 and an R^2^ of 0.423. However, when the data was related to high-water-stress conditions (i.e., after MPP), LR presented the best performance, with an RMSE of 5.52 and an R^2^ of 0.628. Therefore, it is possible to see a clear linear relationship between ISC and leaf mass under advanced dehydration. Indeed, all models presented a linear response pattern, as shown in Figure 4. Regarding FMC estimation, the models do not converge with data collected prior to the MPP. However, GPR again represents the most efficient model, with an RMSE of 0.1138 and an R^2^ of 0.788 under post-MPP conditions. Similarly, MPP achieved close performance, with an RMSE of 0.1288 and R^2^ of 0.787. In all cases, the FMC prediction presented a lower RMSE and greater explanatory power than the mass estimate. These findings disclose that the leaf mass is strongly related to the electrical parameters under high-water-stress conditions. Figure 5 presents the performance evaluation of the regression models for predicting FMC using VOC and ISC, based on data collected after the MPP.

For *Beta vulgaris*, the correlations between FMC and electrical parameters were further analyzed under three validation schemes (k-fold cross-validation, holdout, and resubstitution) to assess model robustness. In the FMC–current case, single-hidden-layer neural networks with ReLU activation consistently achieved the highest or near-highest performance across all schemes, with test R^2^ values between approximately 0.52 and 0.75, accompanied by a relatively low RMSE and MAE. This result indicates that moderately complex nonlinear mappings were sufficient to capture the underlying behavior of the data. Gaussian process regression models disclosed similarly strong performance, with only slightly lower R^2^ values, whereas cubic-kernel SVMs failed to generalize and frequently produced negative R^2^ values and a large RMSE, confirming that this kernel configuration is not appropriate for this dataset. On the other hand, for VOC prediction, SVMs with Gaussian kernels systematically outperformed the other models, reaching test R^2^ values above 0.82 in cross-validation and close to 0.86 in holdout validation, with very small error magnitudes. These findings suggest that the voltage signal encodes water content information in a way that is particularly well captured by smooth, radial basis nonlinear mappings, whereas linear models and efficient linear SVM variants show clear limitations when dealing with the intrinsic nonlinearity of the problem.

In addition, a similar analysis was conducted for fresh mass prediction using current and voltage as input features. In the mass–current case, Gaussian-kernel SVMs and shallow neural networks provided the best balance between accuracy and generalization, achieving test R^2^ values of approximately 0.53–0.55 in cross-validation and holdout. By contrast, linear regression and efficient linear SVM models produced negative R^2^ values and a large RMSE, again demonstrating the inadequacy of purely linear decision functions for this task. For the mass–voltage relationship, ensemble methods and robust linear regression achieved the most stable performance, with R^2^ values approaching 0.67 under resubstitution and between approximately 0.54 and 0.70 in cross-validation and holdout. These results indicate that combinations of weak learners or robust linear fits can effectively exploit the predictive information encoded in the voltage signal. Kernel-based SVMs and multilayer perceptrons remained competitive but did not consistently surpass the best ensemble or robust linear models. Overall, these findings confirm that nonlinear approaches are essential for leveraging the predictive content of EFEH electrical signals, with Gaussian-kernel SVMs, shallow neural networks, and ensemble methods emerging as the most reliable options across traits, input variables, and validation schemes.

### 3.4. Performance Evaluation of Regression Models for *Lactuca sativa*

Table 3 presents the results for the *Lactuca sativa* species. It is worth noting that the several regression models diverged when they were trained with data associated with early stages of dehydration, indicating that no clear correlation can be observed. On the other hand, the analysis of high-dehydration data (i.e., under high water stress) using regression models presented low-performance models. In other words, the data presents high variability that cannot be determined with simple regression models. Therefore, future works will be focused on controlled drying processes according to species (at lower temperatures) to improve data consistency and the performance of models.

For *Lactuca sativa*, the correlations between FMC and electrical parameters were also analyzed under k-fold cross-validation, holdout, and resubstitution schemes. In the FMC–current case, the best test performance under cross-validation was obtained with a single-hidden-layer neural network (10 neurons, ReLU), which achieved a test R^2^ of approximately 0.28 with a low RMSE and MAE, closely followed by standard and robust linear regression models with almost identical metrics. Under holdout validation, linear and Gaussian-kernel SVMs, together with exponential-kernel GPR, provided similar, moderate test R^2^ values (around 0.21–0.22), again confirming that all three methods captured only a limited portion of the variance while maintaining reasonable generalization. In resubstitution, a deeper three-layer neural network slightly outperformed ensemble configurations, with test R^2^ values close to 0.38, whereas cubic-kernel SVMs systematically yielded a negative R^2^ and large RMSE across schemes, indicating a clear mismatch between this kernel configuration and the structure of the FMC–current data. For FMC–voltage, performance remained in a similar range: least-squares kernel models and shallow or three-layer neural networks achieved test R^2^ values around 0.27–0.28 in cross-validation, GPR models with different covariance kernels reached test R^2^ values near 0.24 in holdout, and resubstitution results showed that trees could overfit the training data while generalizing poorly. Overall, these results indicate that, for *Lactuca sativa*, FMC is only weakly encoded in the electrical features, and even the best nonlinear models provide limited but consistent predictive power.

A similar analysis was carried out for fresh mass prediction using current and voltage as input variables in the *Lactuca sativa* subset. For the mass–current relationship, cross-validation experiments showed that linear regression and quadratic- or linear-kernel SVMs achieved the best and very similar test R^2^ values (around 0.31), with moderate errors and good agreement between validation and test sets, whereas efficient linear models again produced a negative R^2^ and much larger RMSE. Under holdout validation, two-hidden-layer neural networks, exponential-kernel GPR, and Gaussian-kernel SVMs offered the most balanced performance, with test R^2^ values close to 0.49–0.51, while cubic-kernel SVMs exhibited poor generalization and large errors. In resubstitution, shallow and two-layer neural networks, together with exponential-kernel GPR, delivered the highest test R^2^ (around 0.42–0.45), confirming the advantage of nonlinear models in this setting. For the mass–voltage relationship, GPR with exponential kernels, least-squares kernel models, and shallow neural networks attained the best performance under cross-validation, with test R^2^ values close to 0.56 and consistent RMSE across validation and test. Holdout and resubstitution results further showed that Gaussian process models, ensembles, and decision trees could provide a reasonably high test R^2^ (up to about 0.52–0.70), albeit with signs of overfitting in some configurations and again with cubic-kernel SVMs performing worst. The *Lactuca sativa* results indicate that, although predictive performance is lower than in the other datasets, current- and voltage-based features still retain useful information about mass and FMC and that nonlinear methods—particularly GPR, SVMs with appropriate kernels, ensembles, and shallow NNs—are better suited than purely linear models for exploiting this information.

### 3.5. Real-World Applications

To evaluate the performance of leafy vegetable-based EFEHs in real-world applications, we analyzed their capabilities as power supplies following the methodology described in [16]. Although EFEHs assembled with *Lactuca sativa* leaves outperform those assembled with *Beta vulgaris* leaves in terms of electrostatic charge collection, only *Beta vulgaris* samples were used due to the practical advantages previously identified.

**Figure 6:**
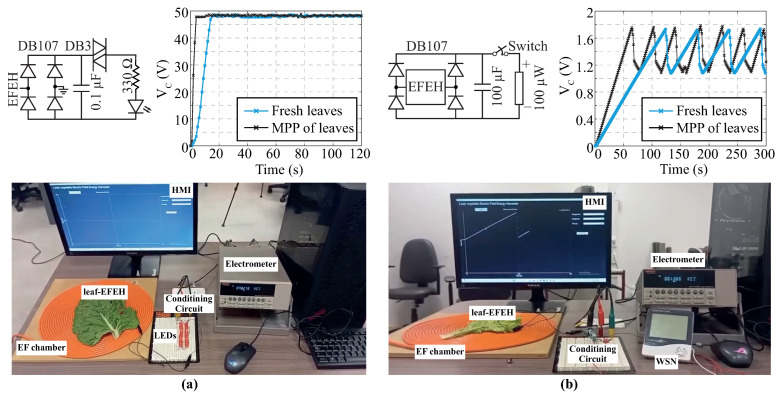
Real-world applications of EFEHs assembled with *Beta vulgaris* samples. (**a**) Powering a 27-LED array using EFEHs under controlled laboratory conditions. (**b**) Powering a 100 μW thermo-hygrometer connected to a capacitor, operating in a low-duty-cycle mode driven through an external electrical switch. Note: The solid cyan line represents the results associated with freshly harvested samples, while the solid black line corresponds to samples dried at 60 °C for 50 min.

The first experiment demonstrates the capability to develop self-powered active indicators capable of providing early warnings related to crop status and health. Therefore, an EFEH was used to power a 27-LED array, driven by a simple switching circuit, as illustrated in Figure 6a. Experimental results revealed a classic capacitor charging profile, where the voltage increased over approximately 16 s until reaching the breakdown voltage of the DB3-diac (approximately 50 V). Once this threshold was reached, the DB3-diac began conducting, enabling the LED array to light up continuously. Next, the leaf was subjected to the drying procedure described in Section 2 for 50 min (empirical time to reach MPP). As expected, the charging time decreased significantly to 4 s. This finding demonstrates an outstanding enhancement in the electrical output of the EFEH under high-water-stress conditions.

As a second experiment, we analyzed whether the EFEH can operate as a power source for low-power electronics, specifically focusing on the activation of wireless sensor nodes. To this end, a low-duty-cycle operation strategy, previously proposed in [34], was adopted to minimize power consumption. A power management circuit was implemented, consisting of a manual switch and a 100 μF capacitor, as illustrated in Figure 6b. The selected load is a 100 μW thermo-hygrometer. When the capacitor is fully discharged, the EFEH charges it to 1.8 V in approximately 123.8 s. The voltage is determined according the thermo-hygrometer operating voltage. By contrast, under high-water-stress conditions, the charging time is reduced to 64.5 s. Once the operating voltage is reached, the sensor is capable of operating and transmitting data every 40.4 s and 24.4 s for fresh and dry samples, respectively. The device operates in a two-second active window, which is sufficient to perform sensing or data transmission tasks, after which it returns to a sleep mode while waiting for the next activation cycle. If a longer operational time is required, the capacitor value can be increased. However, it is important to note that higher capacitance values may lead to increased leakage currents, which can significantly reduce the efficiency of the EFEH system.

In the Supplementary Material (Videos S1 and S2), we provide real laboratory recordings of the experimental procedures for both tests, allowing direct visualization of the EFEH operation and the data collection process

## 4. Discussion

Plant tissues can operate as self-adaptive bio-dielectric elements for EFEH deployments. The vegetable-based EFEH functions as a water content-dependent variable capacitor, in which the electrolyte solution of leaves modulates dielectric polarization and charge collection efficiency. The maximum performance under moderate dehydration exhibits maximum capacitive coupling between the external collector plate and the internal ionic matrix of leaves. After the MPP, ionic mobility collapses, thereby reducing charge transfer and power output. This phenomenon is supported by experimental findings demonstrating that the electrical performance of EFEHs highly depends on leaf water content, which directly influences charge transport and output stability.

The structural and inner traits of the species play an important role in the collected energy. The thicker and more moisture-retentive leaves of *Beta vulgaris* present high stability in ion conduction. On the other hand, *Lactuca sative* presents higher instantaneous output but low repeatability due to its thinner and less structured surface. This detected advantage is particularly important for real-world agricultural applications, where self-powered active sensors need to exhibit high repeatability and long-term stability, rather than merely high measurement capacity.

The outstanding capability of EFEHs to power low-power portable electronics under controlled conditions demonstrate their potential for autonomous agricultural monitoring systems. This ability enables the development of self-powered LED indicators for crop monitoring and early warning applications, thereby contributing to improved agricultural security. Although the proposed EFEHs were evaluated under laboratory conditions, the results confirm their technological feasibility for real-world implementation. Furthermore, the results reveal the potential of EFEHs as sustainable power sources for distributed low-power electronics in agricultural environments—particularly in areas with limited mobility and restricted access, such as greenhouses.

### Comparison with State-of-the-Art Energy Harvesters

This section introduces a comparative analysis of the proposed leaf-based EFEH with representative state-of-the-art energy harvesters used in plant-compatible or low-power agricultural sensing systems. Table 4 summarizes the main performance metrics, including device type, operating principle, reported maximum power, frequency or excitation conditions, and suitability for integration with living plants.

## 5. Conclusions

This work presented the dual role of leaf-based EFEHs as energy harvesting devices and self-powered bio-electronic systems. By assembling EFEHs with hydroponic leafy vegetables, we determined that the electrical output highly depends on the fresh mass and FMC, behaving as a humidity-dependent variable capacitor. Empirical results disclosed that the maximum performance was achieved under moderate dehydration, i.e., optimal capacitive coupling between the external electric field and the internal ionic matrix of the leaf. In contrast, severe drying leads to a collapse of ionic mobility and inhibits charge transfer. Although *Lactuca sativa* achieved up to 10 % higher VOC and ISC, *Beta vulgaris* presented more electrical stability and lower data dispersion, identifying it as the more reliable candidate for long-term self-powered sensor development. These results indicate that the internal leaf traits directly affect electrical parameters of EFEHs. Moreover, regression models disclosed that FCM and fresh mass are reliable predictors of electrical parameters, supporting the feasibility of data-driven phenotyping through EFEHs. Real-world tests demonstrated that leaf-based EFEHs are able to operate as power sources, driving up to 27 commercial green LEDs and low-power portable electronics. These findings demonstrate that EFEHs can function as sustainable, self-powered, and noninvasive indicators of plant physiological state in living leafy vegetable crops.

## Figures and Tables

**Table 1:** Main hyperparameters and configurations used during the training process of the proposed ML regression models.

ML Model	Hyperparameter
LR	Linear terms Iteratively reweighted least squares
GPR	Basis function: Linear Standardize data Kernel function: Nonisotropic Exponential Kernel scale: 0.11823 Sigma: 41.0277
MLP	Number of fully connected layers: 2 Activation function: RELU Number of neurons: 39 and 212 Lambda: 0.054257 Standardize data

**Table 2:** Comparison of the estimation results for two plant functional traits of *Beta vulgaris*—FMC and leaf mass—based on VOC and ISC, using three regression models: Linear regression, Gaussian process regression, and Multilayer perceptron. The model performance is analyzed using R^2^ and RMSE. Note: Bold values indicate the model with the best performance. Empty cells represent models that diverged.

Experiment Analysis	Regression Model	Mass (g)		FMC (%)	
		RMSE	R^2^	RMSE	R^2^
Trait vs. VOC before MPP	LR	16.8400	0.2077	-	-
	GPR	**15.3940**	**0.3380**	-	-
	MLP	16.6420	0.2263	-	-
Trait vs. VOC after MPP	LR	6.5634	0.6789	0.1819	0.4576
	GPR	**6.3696**	**0.6976**	**0.1138**	**0.7877**
	MLP	6.4130	0.6935	0.1155	0.7815
Trait vs. ISC before MPP	LR	**10.9060**	**0.24598**	-	-
	GPR	9.5402	0.4230	-	-
	MLP	10.9060	0.2455	-	-
Trait vs. ISC after MPP	LR	**5.5180**	**0.6275**	0.1924	0.5351
	GPR	5.7359	0.5975	**0.1268**	**0.7939**
	MLP	5.6884	0.6041	0.1288	0.7870

**Table 3:** Comparison of the estimation results for two plant functional traits of *Lactuca sativa*—FMC and leaf mass—based on VOC and ISC, using three regression models: linear regression, Gaussian process regression, and multilayer perceptron. The model performance is analyzed using R^2^ and RMSE. Note: Bold values indicate the model with the best performance. Empty cells represent models that diverged.

Experiment Analysis	Regression Model	Mass (g)		FMC (%)	
		RMSE	R^2^	RMSE	R^2^
Trait vs. VOC before MPP	LR	-	-	-	-
	GPR	**2.3288**	**0.1576**	-	-
	MLP	-	-	-	-
Trait vs. VOC after MPP	LR	**0.8020**	**0.4299**	0.1103	0.1641
	GPR	0.8347	0.3826	0.1103	0.1641
	MLP	0.8151	0.4112	0.1103	0.1641
Trait vs. ISC before MPP	LR	**-**	**-**	-	-
	GPR	-	-	-	-
	MLP	-	-	-	-
Trait vs. ISC after MPP	LR	0.8476	0.4472	0.1130	0.2930
	GPR	0.8483	0.4463	0.1128	0.2930
	MLP	**0.8463**	**0.4490**	**0.1121**	**0.3039**

**Table 4:** Comparison of the proposed leaf-based EFEH with representative state-of-the-art energy harvesters assembled with vegetables.

Work	Material	Operational Mode	Power Density	Application
This work	Beta vulgaris	Electric field	8.45 V; 846 nA at 10 MΩ	- Noninvasive FMC. - Works without motion. - Hydroponics-compatible. - DC output after rectification.
[15]	Leek outer	Triboelectricity	182 V 0.83 mA/m^2^	- High power. - Requires mechanical motion. - Gas/humidity-sensitive. - High-voltage generation. - Environmental sensitivity.
[15]	Onion outer	Triboelectricity	60 V 0.25 mA/m^2^	
[15]	Scallion outer	Triboelectricity	32 V 0.11 mA/m^2^	
[15]	Leek skin	Triboelectricity	300 V 436 μA	
[15]	Onion skin	Triboelectricity	746 V 144 μA	
[15]	Scallion skin	Triboelectricity	315 V 480 μA	
[14]	Lettuce Mustard Celery cabbage	Triboelectricity	0.5 to 2 V 0.15–0.29 μA	- Editable materials. - Low power. - Basic sensors.
[19]	Hosta Magnolia denudata Chinese leaves Populus Lotus Epipremnum	Triboelectricity	90–120 V 2 to 4 μA	- Harvesting leaf vibration - Strongly motion-dependent.

## Data Availability

Pre-processed and processed data will be available upon request.

## Supplementary Materials

The following supporting information can be downloaded at https://www.mdpi.com/article/10.3390/bios16010013/s1, Video S1: Demonstration of LED activation powered by a leaf-based electric field energy harvester (leaf-EFEH). Video S2: Demonstration of temperature and humidity sensor operation powered by a leaf-based electric field energy harvester (leaf-EFEH).
